# Biotechnological Advancements and Begomovirus Management in Okra (*Abelmoschus esculentus* L.): Status and Perspectives

**DOI:** 10.3389/fpls.2017.00360

**Published:** 2017-03-17

**Authors:** Gyan P. Mishra, Bijendra Singh, Tania Seth, Achuit K. Singh, Jaydeep Halder, Nagendran Krishnan, Shailesh K. Tiwari, Prabhakar M. Singh

**Affiliations:** Department of Biotechnology, ICAR-Indian Institute of Vegetable ResearchVaranasi, India

**Keywords:** geminiviruses, infectious clones, ladies' finger, OELCV, recombination, transcriptomics, YVMV

## Abstract

Despite the importance of okra, as one of the important vegetable crop, very little attention has been paid to its genetic improvement using advanced biotechnological tools. The exploitation of marker assisted breeding in okra is often limited due to the availability of a few molecular markers, the absence of molecular genetic-map(s), and other molecular tools. Chromosome linkage-groups were not yet constructed for this crop and reports on marker development are very scanty and mostly hovering around cultivar characterization. Besides, very little progress has been observed for transgenic development. However, high throughput biotechnological tools like chromosome engineering, RNA interference (RNAi), marker-assisted recurrent selection (MARS), genome-wide selection (GWS), targeted gene replacement, next generation sequencing (NGS), and nanobiotechnology can provide a rapid way for okra improvement. Further, the etiology of many deadly viral diseases like the yellow vein mosaic virus (YVMV) and okra enation leaf curl virus (OELCV) in okra is broadly indistinct and has been shown to be caused by various begomovirus species. These diseases cause systemic infections and have a very effective mode of transmission; thus, preventing their spread has been very complicated. Biotechnological interventions have the potential to enhance okra production even under different viral-stress conditions. In this background, this review deals with the biotechnological advancements in okra *per se* along with the begomoviruses infecting okra, and special emphasis has been laid on the exploitation of advanced genomic tools for the development of resistant varieties.

## Introduction

Okra (*Abelmoschus esculentus* L. Moench), belonging to Malvaceae family is originally included in the genus *Hibiscus;* however, section *Abelmoschus* is now accepted as distinct genus on the basis of its caducous nature of the calyx (Dhankhar et al., 2005). The word *Abelmoschus* perhaps originated from the Arabian word “*abul-l-mosk”* meaning “source of musk,” referring to the musky smell of the seeds (Charrler, 1984). This genus is important because of two cultivated species, *A. esculentus* and *A. caillei* (Patil et al., 2015). It is believed to be the native of South Africa and the first recorded reference was by the Egyptians in 1216 A.D. (Lamont, 1999). A putative ancestor (*A. tuberculatus*, 2*n* = 58) being native to Uttar Pradesh in India, suggests the Indian origin whereas, the presence of another putative ancestor (*A. ficulneus*, 2*n* = 72) in East Africa, suggesting northern Egypt and Ethiopia as its geographical origin (Charrler, 1984). Okra is an allopolyploid, having lowest known chromosome number as 2*n* = 56 in *A. angulosus* and the highest around 200 in *A. caillei*, which is an amphipolyploid (allotetraploid) between *A. esculentus* (2*n* = 130–140) and *A. manihot* (2*n* = 60–68) (Siemonsma, 1982). Even within *A. esculentus*, a regular series of polyploids having chromosome numbers 2*n* = 72, 108, 120, 132, and 144 which are derived with a basal *n* = 12 are reported (Datta and Naug, 1968). Of fifty described species, eight are most widely accepted by the scientists working on okra globally (IBPGR, 1991).

More than 99% of okra cultivation is done exclusively in the developing countries of Asia and Africa with very poor productivity, especially in African countries (2.25 MT ha^−1^) compared with any other region. Globally, okra is occupying an area of 1.83 million ha, yielding 9.62 million metric tons (MT) annually having an average yield of 5.26 MT ha^−1^. India ranks first in the world with a production of 6.3 million MT (72% of the total world production) from over 0.5 million ha area with 12.0 MT ha^−1^ productivity (FAOSTAT, 2014).

Among the genus *Abelmoschus, A. esculentus* is most widely cultivated for its pods throughout Asia and Africa. In the West and Central Africa, *A. caillei* is cultivated for leaves and pods (Siemonsma, 1982) whereas; in the South Pacific islands, *A. manihot* is extensively grown for its leaves. *A. moschatus* is grown as an ornamental plant and also for its aromatic seeds. The other species namely, *A. tetraphyllus, A. tuberculatus, A. ficulneus, A. crinitus, A. enbeepeegearense, A. palianus*, and *A. angulosus* are true wild species (Patil et al., 2015). Okra has great potential as foreign exchange earner and accounts for about 60% of the export of fresh vegetables from India to the Middle East and European countries (Singh et al., 2014). It is considered to be an often-cross pollinated crop since insects such as honey bees (*Apis mellifera*) and bumblebees (*Bombus auricomus*) can affect cross-pollination (Lamont, 1999).

Okra is considered as an important constituent for balanced food due to its dietary fibers and amino-acid composition which is rich in lysine and tryptophan (Hughes, 2009). Its fruits are harvested when immature and are commonly consumed as salads, soups, and stews (Salameh, 2014). The roots and stems are used for cleaning the cane-juice during brown-sugar preparation (Shetty et al., 2013). The seeds have also gained much interest as a new oil (30–40%) and protein (15–20%) source (Gemede et al., 2015). It also contains considerable amounts of iron, calcium, manganese, and magnesium, vitamins A, B, C, and K, as well as folates (USDA National Nutrient Database, 2016). It has been found to possess various ethno-pharmacological and medicinal properties against cancer, high-cholesterol, and *Diabetes mellitus* (Jenkins et al., 2005; Sabitha et al., 2011).

Cultivated okra is mostly susceptible to a large number of begomoviruses having overlapping host range, like radish, tomato, cotton etc. Yellow vein mosaic disease (YVMD), okra leaf curl disease (OLCD), and okra enation leaf curl disease (OELCD) are caused by viruses of genus Begomovirus (family Geminiviridae) resulting in the serious losses in okra cultivation (Venkataravanappa et al., 2013b). Under field conditions, infected plants were found to be associated with heavy infestations of the whitefly *Bemisia tabaci*, the vector of begomoviruses (Venkataravanappa et al., 2015a). The loss in yield, due to YVMV and/or OELCV in okra was found ranging from 30 to 100% depending on the age of the plant at the time of infection (Singh, 1996).

**Figure 1:**
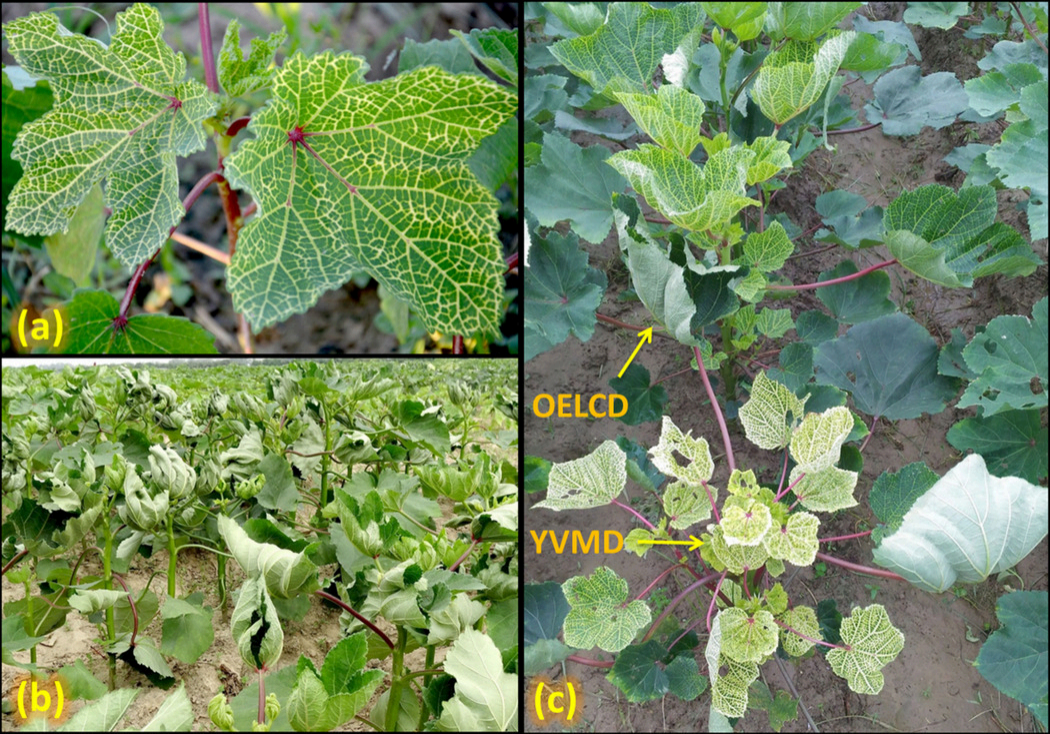
**Okra plants infected with (a)** YVMV, **(b)** OELCV, and **(c)** both YVMV and OELCV diseases.

The YVMD was first reported in 1924 from India (Kulkarni, 1924); is caused by YVMV and subsequently named as “yellow vein mosaic of okra” (Uppal et al., 1940). It is characterized by different degrees of chlorosis and yellowing of veins and veinlets, smaller leaves, fewer and smaller fruits, and stunting (Venkataravanappa et al., 2012a) (Figure 1). Infection of 100% plants in a field is quite usual with yield loss ranging between 50 and 94% (Fajinmi and Fajinmi, 2010).

In India, OELCD was first reported from Bangalore (Karnataka) during the early 1980s; causes yield loss up to 80–90% (Singh, 1996). The characteristic symptoms of OELCD include leaf-curling, vein-thickening, and a decrease in the leaf surface area. Moreover, the infected plants become severely stunted with fruits being small, deformed, and unfit for marketing (Sanwal et al., 2014) (Figure 1). This disease is going to be the future menace of okra cultivation and needs a strategic breeding program to evolve resistance against OELCV (Singh et al., 2013). In the recent past, frequent breakdown of the viral disease resistance has been observed in popular okra varieties like Parbhani Kranti, P-7, Arka Anamika, Arka Abhay (Sanwal et al., 2014). The premise of the evolution of new viral strains seems one of the major factors responsible for the breakdown of tolerance, as the tolerance in the majority of the cases is location specific.

Further, the emergence of polyphagous “B” biotype of *B. tabaci*, mixed cropping system, along with the increased host range of more than 600 plant species has also resulted in geminiviruses infecting previously unaffected crops (Singh et al., 2013; Sanwal et al., 2016). Moreover, OLCV can be transmitted to several weeds and plant species like *Amaranthus retroflexous, Malva parviflora, Gossypium barbadense, Lycopersicon esculentum*, and *Nicotiana tabacum* (Ghanem, 2003). Unlike fungicides and bactericides, no commercial viricides have yet been developed; therefore, viral diseases are not amenable to control by any direct methods (Thresh, 2006).

Exploitation of biotechnology in okra breeding is often limited, due to the availability of a few polymorphic molecular markers and absence of molecular genetic maps. Linkage groups are not yet known, and the situation becomes more complicated due to a large number of chromosomes (2*n* = 56–196) and complex polyploidy nature of the okra genome (Sastry and Zitter, 2014). In this backdrop, this review summarizes the current status of both, biotechnological developments and begomovirus management in okra across the world, and its future improvement strategies.

## Genetic diversity studies using molecular tools

In spite of its high economic value, little attention has been paid to assess the genetic diversity of okra at the molecular level (Fougat et al., 2015). Various reports observed wide phenotypic variability within *A. esculentus*, which still needs molecular confirmation (Kaur et al., 2013; Amoatey et al., 2015). Moreover, the SDS-PAGE analysis was found effective in systematic studies of okra (Osawaru et al., 2014). Reports about the use of molecular markers in okra are very limited, which again were limited to RAPD, ISSR, AFLP, SSR, and SRAP markers for cultivars and germplasm characterization.

Genetic diversity studies using 13 isozymes and 22 RAPDs in *A. esculentus* and the four wild species, revealed moderate genetic diversity within *A. ficulneus*, A. *moschatus*, and *A. esculentus*, whereas *A. moschatus* was observed to be quite distinct from others. Further, gene-duplication was also observed in okra (Bisht et al., 1995). RAPD analysis revealed significantly high genetic diversity between *A. esculentus* and *A. callei* (Aladele et al., 2008). Simple sequence repeats (SSRs) developed for *Medicago truncatula* were successfully used in okra cultivars of Burkina Faso (Sawadogo et al., 2009) for biodiversity studies (Table S1).

Genetic diversity studies among 29 Nigerian okra accessions using RAPD markers identified the most divergent genotypes and their origins (Nwangburuka et al., 2011). The genetic distinctiveness of 44 Indian okra genotypes using 14 RAPD primers revealed two clear groups (Prakash et al., 2011); whereas, considerable genetic variability was recorded in the 39 genotypes of Pakistan when studied using 20 RAPD primers (Haq et al., 2013). Kaur et al. (2013) studied the genetic diversity of 70 okra lines of India and Brazil, using 40 RAPDs and eight morphological traits, and did not found any specific pattern, suggesting independent clustering of the entries.

AFLP analysis of 21 landraces of Jordan revealed some correspondence with the site of germplasm collection (Akash et al., 2013). Another AFLP analysis of 50 okra genotypes from different countries revealed low level (12%) of polymorphism and distinct geographical groupings (Kyriakopoulou et al., 2014). Similar results were also observed by Younis et al. (2015) for 29 Egyptian okra accessions when analyzed using 42 ISSR and 5 AFLP primers. Yildiz et al. (2015) analyzed genetic diversity of 66 okra landraces using 83 inter-primer binding site (iPBS)-retrotransposon markers. Considering the importance of okra, the biodiversity studies reported from different parts of the world seems just a humble beginning (Table 1). Intensive biodiversity studies are required, using global okra germplasm including its wild relatives, for its effective utilization in ongoing breeding programs across the world.

**Table 1:** **Genetic diversity studies in okra using various molecular markers**.

**S. No**.	**Marker**	**Remarks**	**References**
1.	RAPD	*A. esculentus, A. ficulneus, A. manihot, A. moschatus*, and *A. tuberculatus*	Bisht et al., 1995
2.	RAPD	*Abelmoschus* spp.	Martinello et al., 2001
3.	RAPD	42 *Abelmoschus* and 1 *Hibiscus* accessions from different countries	Martinello et al., 2003
4.	SRAP	Turkish germplasm	Gulsen et al., 2007
5.	RAPD	W. African (*A. callei*) and Asian (*A. esculentus*) genotypes	Aladele et al., 2008
6.	SSR	20 Burkina Faso accessions	Sawadogo et al., 2009
7.	RAPD	29 Nigerian *A. esculentus* accessions	Nwangburuka et al., 2011
8.	RAPD	44 okra genotypes from different parts of India	Prakash et al., 2011
9.	SSR	65 accessions of three species	Schafleitner et al., 2013
10.	AFLP	21 Jordan landraces	Akash et al., 2013
11.	RAPD	70 okra lines from India and Brazil	Kaur et al., 2013
12.	RAPD	39 okra genotypes of Pakistan	Haq et al., 2013
13.	ISSR	24 Chinese genotypes	Yuan et al., 2014
14.	AFLP	Landraces from different countries	Salameh, 2014
15.	SDS-PAGE	African germplasm	Osawaru et al., 2014
16.	AFLP	50 okra genotypes from Greece, Argentina, Brazil, India, Cameroon, Chad, China, Turkey, USA, Zambia, and Cote D'Ivoire	Kyriakopoulou et al., 2014
17.	ISSR and AFLP	29 Egyptian accessions	Younis et al., 2015
18.	SSR	24 Indian accessions	Fougat et al., 2015
19.	iPBS-retrotransposons and SSRs	66 okra varieties from America, India, Africa, and Japan	Yildiz et al., 2015

## Optimization of DNA isolation protocols

Okra is such a genomically orphan crop that, even today, many labs across the world are struggling with optimization of quality DNA isolation protocol which can be effectively used for the high-end genomic studies. The main obstacle preventing the extraction and purification of DNA from green okra leaves is the presence of large amounts of mucilaginous acidic polysaccharides, having polygalacturonic acid as its main component (Ahmed et al., 2013). It is observed that, during cell lysis, the nucleic acids come in contact with these polysaccharides and oxidized form of polyphenols binds to the proteins and nucleic acids, resulting in a brown gelatinous material, reducing both yield and purity of extracted DNA (Aljanabi et al., 1999). Furthermore, DNA which dissolves even in the presence of these polysaccharides inhibits various biotechnological activities including restriction digestion, PCR, or *in-vitro* labeling (Sahu et al., 2012). In this backdrop, a large number of researchers have optimized various DNA isolation protocols which are presented in Table S2. Overall, good quality DNA can be obtained in okra, with the caution that it should be done using the proper sample and DNA isolation protocol.

## Transcriptome analysis

Transcriptome analysis has emerged as a powerful tool to obtain gene sequences and develop molecular markers, especially in less researched species or non-model crops including okra (Strickler et al., 2012; Bosamia et al., 2015). RNA sequencing using combined leaf and pod transcriptome of *Abelmoschus esculentus* resulted in approximately 46 m bp data which yielded more than 150,000 unigenes. From these sequences, 935 non-redundant SSR motifs were identified, of which 199 were used for testing in a germplasm set and 161 were found polymorphic (Schafleitner et al., 2013).

Schafleitner et al. (2013) found relatively small unigene size and the low number of full-length cDNAs, which indicated that the sequencing depth was not sufficient to represent the whole transcriptome. Therefore, deeper sequencing of the transcriptome is required not only to enrich the unigene set with full-length transcripts but also to reduce the redundancy of the annotation of the unigenes. However, a certain level of annotation redundancy could also be due to the allopolyploidy of *Abelmoschus*, where transcripts from different genomes with slightly different sequences are present in the transcriptome.

Correct *de-novo* assembly of short sequence reads of polyploid organisms like okra is complex due to the larger size of the transcriptome (Gruenheit et al., 2012). Although RNA sequencing is relatively cheap, but limited funds available for research on genomically orphan crops such as okra, restrains the ability for deep sequencing of the transcriptome, leading to short unigenes representing only partial gene sequences. Therefore, deep-sequencing will result in long unigenes, representing full-length gene sequence, which will be more useful for further biotechnological studies.

## Transformation protocols and transgenic development in okra

The success of utilizing technologies, such as transformation and somaclonal variation in any crop including okra, largely rely on efficient *in vitro* regeneration techniques (Patel et al., 2016). But, the absence of an efficient transformation system has hampered the progress in okra genetic engineering research. Regarding *in vitro* culture of okra, only limited numbers of protocols were reported for shoot organogenesis (Table 2). Tissue culture-based direct shoot regeneration from cotyledon and cotyledonary node explants (Mangat and Roy, 1986), and regeneration of okra plants from callus tissue derived from cotyledonary axil (Roy and Mangat, 1989) are known. Further, Ganesan et al. (2007) have reported plant-regeneration protocol through somatic embryogenesis from suspension culture; while Anisuzzaman et al. (2008a) developed a protocol for mass *in vitro* propagation using meristem culture for disease-free plant production. Anisuzzaman et al. (2008b) have optimized a viable protocol for indirect shoot organogenesis of okra, from leaf-disc and hypocotyl via callus phase. The details of different explants and media composition as reported by various workers for okra transformation are mentioned in Table 2.

**Table 2:** **Details of explants and media composition used for okra transformation**.

**S. No**.	**Explant**	**Media composition**			**References**
		**Morphogenic/Embryogenic callus induction**	**Shoot regeneration (SR)/Embryoids development (ED)**	**Root formation/Embryo into plantlets conversion**	
1.	Node and shoot tip explants	MS medium containing IAA (0.2 mg L^−1^) and 2,4-D (0.2 rag L^−1^) (Morphogenic)	MS medium supplemented with NAA (1.0 rag L^−1^) and BA (1.0 rag L^−1^) (SR)	MS medium containing NAA (1.0 rag L^−1^)	Mangat and Roy, 1986
2.	Cotyledonary axil	MS medium supplemented with benzyladenine (BA) (1.0 mg L^−1^) (Morphogenic)	BA-enriched MS media containing various concentrations of silver nitrate (0–40 mg L^−1^) (SR)	MS medium containing BA (1.0 mg L^−1^) and NAA (1.0 mg L^−1^)	Roy and Mangat, 1989
3.	Hypocotyls	Medium containing 0.1–0.3 mg L^−1^ BA and 1–3 mg L^−1^ NAA (Morphogenic)	MS medium supplemented with 1–3 mg L^−1^ benzyladenine (BA) and 0.1–0.3 mg L^−1^ alpha -naphthaleneacetic acid (NAA) (SR)	Medium containing 1.0 mg L^−1^ BA and without auxin	Haider et al., 1993
4.	Hypocotyl	MS salts, Gamborg (B5) vitamins, 2.0 mg dm^−3^ 2,4-dichlorophenoxyacetic acid (2,4-D), 1.0 mg dm^−3^ naphthaleneacetic acid (NAA), 25 mg dm^−3^ polyvinylpyrrolidone, and 30 g dm^−3^ sucrose (Embryogenic)	Suspension culture containing MS salts, B5 vitamins, 2.0 mg dm^−3^ 2,4-D and 1.0 mg dm^−3^ kinetin (ED)	½ MS salts, B5 vitamins, 0.2 mg dm^−3^ benzylaminopurine (BAP) and 0.2 mg dm^−3^ gibberellic acid (GA3) (Embryo to plantlets conversion)	Ganesan et al., 2007
5.	Hypocotyl	MS medium supplemented with 2.0 mg L^−1^ NAA + O.S mg L^−1^ TDZ (Morphogenic)	2.0 mg L^−1^ BAP + 0.1 mg L^−1^ IBA (SR)	1.5 mg L^−1^ NAA	Anisuzzaman et al., 2008b
6.	Shoot tip	MS medium containing 1.0 mg L^−1^ of BAP (Morphogenic)	1.0 mg L^−1^ + 0.5 mg L^−1^ GA_3_ (SR)	1.0 mg L^−1^ IBA	Anisuzzaman et al., 2008b
7.	Hypocotyl	MS medium containing 0.5 mg L^−1^ BAP and 2.0 mg L^−1^ NAA (Morphogenic)	Combination of 2.0 mg L^−1^ BAP + 0.1 mg L^−1^, IAA and 2.0 mg L^−1^ BAP + 0.5 mg L^−1^ NAA (SR)	MS medium containing 2.0 mg L^−1^ IBA	Kabir et al., 2008
8.	Cotyledonary nodal meristem	MS basal medium supplemented with 1.0 mg L^−1^ BAP + 1.0 mg L^−1^ NAA + 0.04 mg L^−1^ TDZ	MS basal medium supplemented with 1.0 mg L^−1^ BAP + 1.0 mg L^−1^ NAA + 0.04 mg L^−1^ TDZ	MS basal medium supplemented with 1.0 mg L^−1^ BAP + 1.0 mg L^−1^ NAA + 0.04 mg L^−1^ TDZ	Mallela et al., 2009
9.	Shoot tip	Combination of 1.0 mg L^−1^ IBA + 0.5 mg/L NAA (Morphogenic)	MS medium supplemented with kinetin 0.5 mg L^−1^	MS medium containing 0.5 mg L^−1^ IAA and 1.0 g activated charcoal	Dhande et al., 2012
10.	Zygotic embryo	–	MSB1 (MS salts, B5 vitamins, agar 0.8%, sucrose 3.0%, kanamycin 50 mg L^−1^, cefotaxime 500 mg L^−1^) (SR)	MSB medium	Narendran et al., 2013

Okra is known to be highly recalcitrant to *Agrobacterium*-mediated genetic transformation and regeneration. However, Narendran et al. (2013) have standardized an okra tissue culture protocol using zygotic embryo explants, and its integration with *Agrobacterium*-mediated transformation. The transgenic Bt plants showed resistance to the okra shoot and fruit borer (*Earias vittella*). Recently, Manickavasagam et al. (2015) have established a tissue culture-independent genetic transformation system for okra using seed as an explant. *Agrobacterium tumefaciens* harboring the binary vector pCAMBIA 1301–bar was used for the transformation and 18.3% transformation efficiency was recorded. There seems an urgent need to shift the research focus to develop more transgenics using coat protein (CP) genes, imparting resistance to the various viral diseases like YVMV and OELCV.

## Begomoviruses infecting okra and need of biotechnological interventions

The Begomoviruses of family Geminiviridae have geminate-particle morphology, circular ssDNA encapsidated in twinned quasiisometric particles (Venkataravanappa et al., 2015a), transmitted by *B. tabaci* and infect many dicotyledonous plants (Seal et al., 2006) (Table 3). Okra is known to be susceptible to at least 27 begomoviruses; of which, Yellow Vein Mosaic Disease (YVMD) and Okra Enation Leaf Curl Disease (OELCD) most severely affect its production in terms of yield and fruit quality (Swanson and Harrison, 1993; Sanwal et al., 2014).

**Table 3:** **List of Begomoviruses affecting okra as reported from various parts of the world**.

**S. No**.	**Begomovirus**	**Country**	**Remarks**	**References**
1.	Bhendi yellow vein mosaic virus (BYVMV)	India	Monopartite	Kulkarni, 1924
2.	Okra Enation Leaf Curl Virus (OELCV)	Nigeria	Monopartite	Atiri, 1984
3.	Okra Leaf Curl Virus (OLCV)	Côte d'Ivoire	Monopartite	N'guessant, 1991
4.	OELCV	Côte d'Ivoire	Monopartite	N'guessant et al., 1992
5.	OLCV	West Africa	Monopartite	Swanson and Harrison, 1993
6.	OLCV	Burkina Faso	Monopartite; occur on wild-host like *Sida acuta*	Konate et al., 1995
7.	Okra enation leaf curl Virus (OELCuV)	India	Monopartite	Singh, 1996
8.	Okra yellow vein mosaic virus (OYVMV)	Pakistan	Monopartite	Zhou et al., 1998
9.	BYVMV	Pakistan	Monopartite	Zhou et al., 1998
10.	OLCV	Saudi Arabia	Monopartite	Ghanem, 2003
11.	BYVMV	India	Monopartite	Jose and Usha, 2003
12.	Okra yellow crinkle virus (OYCrV)	Bamako, Mali	Monopartite	Shih et al., 2007
13.	Sida micrantha mosaic virus	Brazil	Monopartite	Fauquet et al., 2008
14.	OYCrV	Mali and Cameroon	Monopartite	Kon et al., 2009
15.	Okra yellow mosaic Mexico virus (OYMMV)	Mexico	Monopartite	Hernandez-Zepeda et al., 2010
16.	Okra yellow mottle Igula virus (OKYMoIV)	Mexico	Monopartite	Hernandez-Zepeda et al., 2010
17.	OELCuV	India	Beta satellite	Venkataravanappa et al., 2011
18.	Bhendi yellow vein Maharashtra virus	India	Monopartite	Brown et al., 2012
19.	Bhendi yellow vein Delhi virus (BYVDeV)	India		Venkataravanappa et al., 2012a
20.	Bhendi yellow vein Haryana virus	India	Monopartite	Brown et al., 2012
21.	Radish leaf curl virus	India	Monopartite	Kumar et al., 2012
22.	Cotton leaf curl Alabad virus (CLCuAV)	India	Monopartite	Venkataravanappa et al., 2012b, 2013a
23.	Okra leaf curl Cameroon virus (OLCuCMV)	Cameroon	Monopartite	Leke et al., 2013
24.	Cotton leaf curl Bangaluru virus (CLCuBaV)	India	Monopartite	Venkataravanappa et al., 2013a
25.	Bhendi yellow vein Bhubhaneswar virus (BYVBhV)	India	Monopartite	Venkataravanappa et al., 2013b
26.	OELCuV	India	Monopartite	Singh et al., 2013
27.	OELCV	India	Monopartite	Sanwal et al., 2014
28.	Okra leaf curl disease-associated DNA 1, isolate OBKG (OLCuA)	India	Alpha satellite	Chandran et al., 2013
29.	Okra yellow crinkle Cameroon alphasatellite (OYCrCMA)	India	Alpha satellite	Chandran et al., 2013
30.	OELCuV	India	Alpha satellite	Chandran et al., 2013
31.	BYVMV	India	Monopartite	Venkataravanappa and Reddy, 2013
32.	Okra leaf curl Oman virus (OLCOMV)	Oman	Monopartite	Akhtar et al., 2014
33.	OELCuV	Pakistan	Monopartite	Hameed et al., 2014
34.	OELCuV complex	Pakistan	Alpha satellite	Serfraz et al., 2014
35.	Cotton leaf curl Gezira virus (CLCuGeV)	Sudan	Monopartite	Venkataravanappa et al., 2014
36.	OLCV	India	Monopartite	Sayed et al., 2014
37.	Okra mosaic virus disease (OMVD) and okra leaf curl disease (OLCD)	Nigeria	Monopartite	Sergius and Esther, 2014
38.	Bhendi yellow vein Madurai virus	India	Monopartite, captured DNA-B of ToLCNDV	Venkataravanappa et al., 2014, 2015b
39.	OELCuV	India	Monopartite	Venkataravanappa et al., 2015a

**Figure 2:**
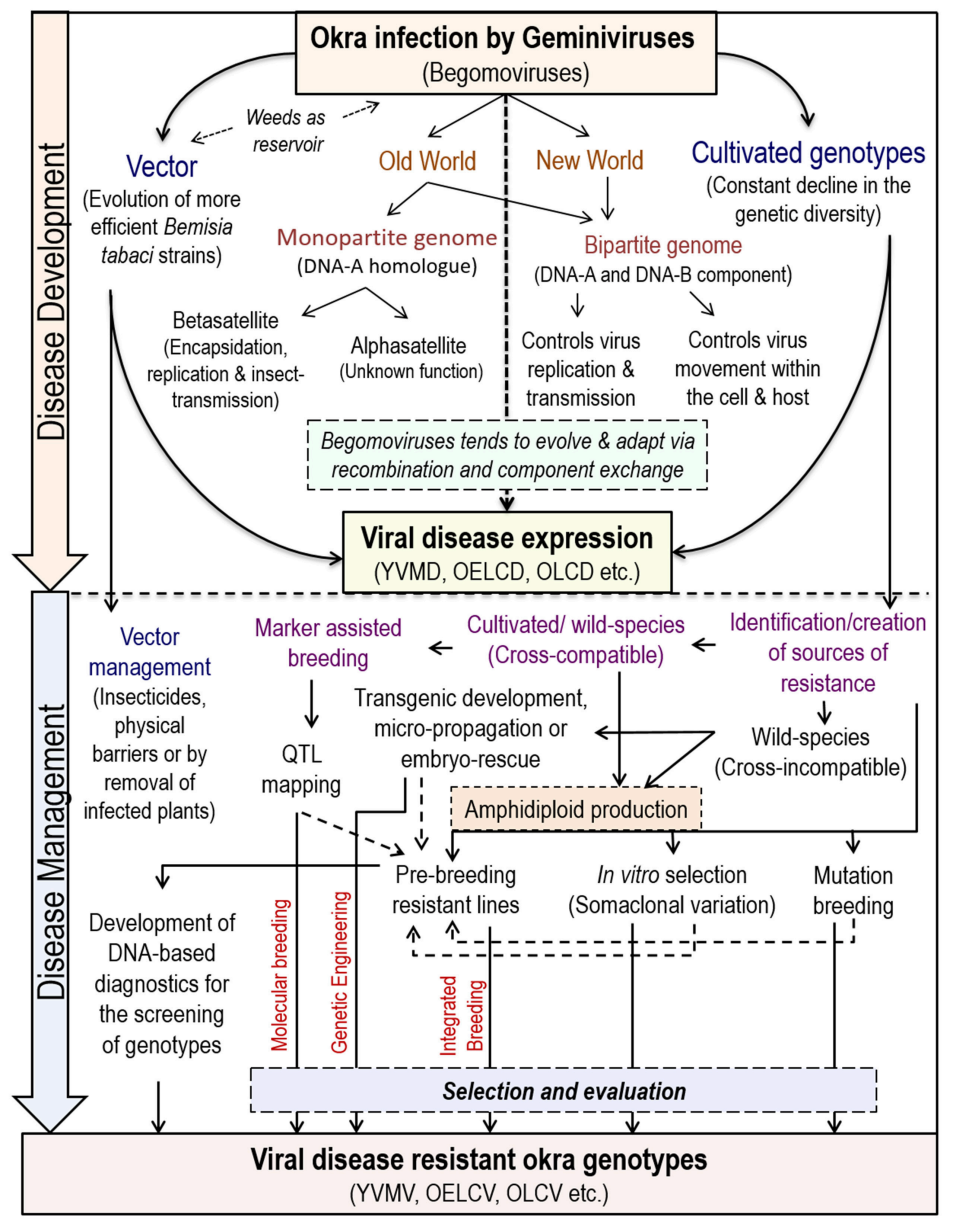
**Begomovirus induced disease development and strategies of its management in okra**.

The begomoviruses native to the New World have only bipartite genomes (having DNA-A and DNA-B components) whereas, of Old World have both bipartite and monopartite (have DNA-A homolog and lacks DNA-B) genomes (Brown et al., 2012) (Figure 2). The DNA-A component is capable of autonomous replication; encodes factors required for the viral encapsidation, replication, and suppression of host-defense. Whereas, DNA-B encodes factors essential for viral systemic movement, host-range determination and symptom expression in host-plants (Rojas et al., 2005).

The monopartite begomoviruses are more numerous than the bipartite types, and are often associated with a class of ssDNA molecules known as betasatellite or DNA-b (1,350 bp) (Saunders et al., 2000), which is required for the symptom-induction, host-range determination, replication, encapsidation, insect-transmission, and movement in the plants (Briddon and Stanley, 2006). Further, minimum 16 types of begomoviruses and 4 types of betasatellites are found associated with the YVMD in different combinations (Singh et al., 2013). Like DNA-B, these satellites depend upon the DNA-A component for encapsidation, replication, and insect transmission (Briddon et al., 2004).

In some cases, the begomoviruses are also found associated with small ssDNA molecules (ca 1.4 kDa), or alphasatellites, which encodes replication rolling cycle initiator- protein (Rep); but need helper virus for infection, encapsidation, and vector-transmission. Moreover, the exact role of alphsatellites is still unknown (Briddon and Stanley, 2006). However, RNAi silencing suggested its probable role in overcoming the host-defense (Nawaz-ul-Rehman et al., 2010). An alphasatellite DNA of 1,350 nucleotides was found associated with OELCuV in okra and showed 79.7% similarity to Hollyhock yellow vein virus-associated symptomless alphasatellite (HoYVSLA) (Chandran et al., 2013).

Most of the begomoviruses characterized from okra across the world are monopartite (Jose and Usha, 2003). However, a bipartite begomoviruses, tomato leaf curl New Delhi virus (ToLCNDV), and a bipartite species closely related it having bhendi yellow vein Delhi virus (BYVDeV), were isolated from okra (Venkataravanappa et al., 2012a). In addition, various evidence suggested that bhendi yellow vein mosaic virus (BYVMV) has adapted co-infection either with BYVB or with the DNA-B-like sequence of ToLCNDV (Venkataravanappa et al., 2015b).

## Recombination and evolutionary divergence of begomoviruses infecting okra

The wide diversity among begomoviruses associated with mixed infections is supposedly assisting in recombination and pseudo-recombination events leading to the frequent emergence of novel begomoviruses, having devastating effects on the okra (Padidam et al., 1999). Recombination has played a significant role in the evolution of geminiviruses (Seal et al., 2006) including the origin of OELCuV; as the sequences making up OELCuV have originated from other malvaceous begomoviruses; cotton leaf curl Bangaluru virus (CLCuBaV), mesta yellow vein mosaic virus (MeYVMV), and BYVMV (Venkataravanappa et al., 2015a). It is reported that OELCuD may be caused by OELCuV in association with at least two distinct betasatellites viz. Bhendi yellow vein betasatellite (BYVB) and Okra leaf curl betasatellite (OLCuB) (Venkataravanappa et al., 2015a).

The recombination analysis among okra leaf curl betasatellites (Hyderabad, India) and BYVMV associated betasatellite showed six major and minor interspecific recombination events, hot-spots and breakpoints (Sohrab et al., 2015). Whereas, recombination analysis of Okra yellow vein Bhubhaneswar virus (OYBHUV) suggested its probable origin due to the exchange of genomic segments between Croton yellow vein mosaic virus (CYVMV), BYVMV, Cotton leaf curl multan virus (CLCuMuV), and MeYVMV (Venkataravanappa et al., 2013b). Although, CLCuBaV was first identified in cotton (Chowda-Reddy et al., 2005), but its detailed characterization suggested it as an okra virus, occasionally infecting cotton (Venkataravanappa et al., 2015a). A betasatellite of YVMD of okra was identified in the Indian samples in which Koch's postulate has been proved by agro-inoculation of BYVMV and bhendi yellow vein betasatellite (BYVB) (Jose and Usha, 2003). Sequence comparisons of begomoviruses from various locations also revealed the existence of different species having several recombinant genome fragments (Venkataravanappa et al., 2014) (Table S3).

Generally, in southern parts of India YVMV and OLCV diseases of okra show either leaf curl or yellow vein mosaic symptoms (Sohrab et al., 2013). However, under Northern Indian conditions, we have observed both YVMV and OELCV symptoms together on the same plants (Figure 1c); which could be due to the emergence of new viral strains due to the recombination or pseudo-recombination. Thus, screening of breeding populations should be planned in these hotspot areas (Sanwal et al., 2014). Further analysis using infectious clones are required to decipher the contribution of individual components viz. virus and betasatellite (Venkataravanappa et al., 2015a).

## Rolling circle amplification (RCA) and infectious clones as a tool for the begomoviruses identification

RCA technique employs a bacteriophage and phi-29 DNA polymerase for the construction of infectious clones of geminivirus and also for the diagnosis, cloning, and genomics of geminiviruses (Inoue-Nagata et al., 2004; Haible et al., 2006). This technique does not require sequence information for cloning and is less expensive and time-consuming than conventional PCR-based methods (Wu et al., 2008; Bang et al., 2014). The amplification products can be sequenced directly (Jeske et al., 2010) or used for the cloning of geminiviral genomes (Inoue-Nagata et al., 2004; Wu et al., 2008) and biolistic inoculation (Jeske et al., 2010; Aranha et al., 2011).

Aranha et al. (2011) have demonstrated the infectivity of rolling circle amplified DNA to okra, *Sida santaremnensis* and to a group of Solanaceae plants after its insertion through biolistics. Kon et al. (2009) have confirmed that the OYCrV and CLCuGV are monopartite begomoviruses and OLCD is caused by a begomovirus/satellite complex. Moreover, typical OLCD symptom was observed only when a betasatellite was co-inoculated with the infectious clones of OYCrV or CLCuGV. Using RCA, Kumar et al. (2012) have constructed the infectious clones and presence of identical viral DNAs were confirmed by sequencing. Further, infectivity testing of tobacco and okra plants using a mixture of begomovirus and alpha and betasatellite infectious clones resulted in typical viral symptoms. This confirmed that the RaLCV and its associated Satellites as the causal agent of okra leaf curl disease. The RCA-based construction of various infectious viral clones can be very useful to other emerging begomoviruses of okra. Further, re-circularization of cloned viral insert was an easy and fast method to produce infectious clones, which can facilitate the fulfillment of “Koch's Postulates” for begomoviruses (Aranha et al., 2011).

## Genetics of begomovirus resistance in okra

Among various diseases caused by the begomoviruses in okra, the inheritance of only YVMV disease has been reported by various researchers (Table 4). Since 1962, there are reports regarding its genetics which are quite contradictory, this could be attributed to the complex nature of the disease and also because of vector establishment based on environmental conditions (Shetty et al., 2013). Among wide relatives, *Abelmoschus angulosus* showed complete resistance to YVMV and two complimentary genes controlling YVMV resistance is observed whose source is either wild ssp. *manihot* or the symptomless carrier genotype, IC1542 (Dhankhar et al., 2005). Since various begomovirus infecting cotton such as CLCuBaV, CLCuMuV, CLCuVBur3, and CLCGV were found infecting okra too, therefore, we have also analyzed the genetics of begomoviruses infecting cotton, which belong to the same Malvaceae family. A range of genetic control like two dominant and a supplementary gene (Wilson and Brown, 1991) for Cotton cultivar Cedix to cotton leaf crumple virus (CLCrV); presence of at least five factors (Khan et al., 2007) for Cotton leaf curl virus (CLCuV); and involvement of three genes, two for resistance and one imparting suppression of the resistance (Rahman et al., 2005) for CLCuD have been observed. Further, Ahuja et al. (2007) reported duplicate dominant, dominant inhibitory, duplicate recessive epistasis and triplicate dominant epistasis for CLCuD, in 22 cross combinations. As the information pertaining to okra infecting begomoviruses are found limited to YVMV, hence, research should be targeted to find the genetic basis of resistance to other begomoviruses infecting okra, under different environmental conditions and in different backgrounds including wild species.

**Table 4:** **Genetics of YVMV resistance in okra**.

**S. No**.	**Resistant parent (R)**	**Susceptible parent (S)**	**Cross**	**Gene action/ Remarks**	**References**
1.	*A. manihot* (L.) Medik and *A. manihot* (L.) Medik ssp. manihot	*A. esculentus* cv. Pusa Sawani	F_2_, BC, and subsequent generations	Single dominant gene	Jambhale and Nerkar, 1981
2.	*A. manihot* ssp. manihot	–	Different generations	Two dominant genes	Sharma and Dhillon, 1983
3.	*A. manihot*	*A. tetraphyllus*	Different generations	Single dominant gene	Dutta, 1984
4.	Arka Anamika, Punjab Padmini and Arka Abhay	Pusa Sawani, Local and Pusa Makhmali	SxS, SxR	Two complementary dominant genes	Pullaiah et al., 1998
			RxR	Two duplicate dominant genes	
5.	IPSA Okra 1	Parbhani Kranti, SL-44 and SL-46	F_2_ and BC generations	Quantitative with two major factors dependent on gene dosage having incomplete dominant gene action	Ali et al., 2000
6.	Many genotypes	Many genotypes	Grafting tests	Resistance is genetic and not due to escape	Ali et al., 2000
7.	Parbhani Kranti	Punjab 8, Punjab Padmini, Pusa Makhmali, and Pusa Sawani	Nine generations	Additive gene effects more significant than dominance gene effects	Vashisht et al., 2001
8.	*A. manihot* (L.) Medikus ssp. manihot	*A. esculentus* cv. Hisar Unnat	Different generations	Two complimentary dominant genes	Sharma and Sharma, 1984; Dhankhar et al., 2005
9.	Punjab-8 and Parbhani Kranti	Pusa Sawani and Pusa Makhmali	F_2_ and back cross	Both single dominant gene and some minor genes; presence of additive gene effects and duplicate epistatic gene action	Arora et al., 2008
10.	BCO-1 and VNR Green	Pusa Sawani and Arka Anamika	Six generations (P_1_, P_2_, F_1_, F_2_, BC_1_, BC_2_) of Tolerant × Tolerant (T × T), Tolerant × Susceptible (T × S) and Susceptible × Susceptible (S × S) crosses	Two duplicate dominant genes in T × T, and 02 complementary dominant genes in T × S cross	Seth et al., 2017

## Whitefly as vector and factors influencing begomovirus incidence in okra

Whitefly causes heavy damage to the okra, both by direct loss of plant vitality by feeding on cell-sap and also by transmitting the various viral diseases. Geminiviruses have a circulative, non-propagative mode of transmission with the associated latent period since virus need to pass through the insect's gut to the salivary glands (Gray and Banerjee, 1999). Transmission efficiency of begomoviruses varied with the sex of whiteflies and generally, females transmit with greater efficiency than males (Czosnek et al., 2001). The reason for this still remains unclear. Begomoviruses can also be transmitted by grafting; but, seed-transmission or transmission through mechanical inoculation has not yet been established (Brown et al., 2012). Weeds have been reported in many instances as reservoirs of both viruses and vectors (Duffus, 1971). The B-biotype has altered the epidemiology of many begomoviral diseases in okra by introducing different begomoviruses into many new plant species (Sanwal et al., 2016). Thus, infection under field conditions seems dependent exclusively on vectors, which was also supported by the direct correlation recorded between disease incidence and vector populations (N'guessant et al., 1992).

## Management of begomoviruses in okra and way forward

Some of the major challenges for the worldwide okra production include quick evolution and recent spread of begomoviruses, the emergence of novel viral strains and increasing abundance of viruliferous whitefly vectors (Venkataravanappa et al., 2015a). Both conventional and non-conventional or genomic approaches are being applied for the management of this menace which is thoroughly discussed in this section.

In the conventional approaches, the essential prerequisite for improving disease resistance is the availability of a suitable source of resistance (Dhankhar et al., 2005). But, due to the emergence of different viral strains (Venkataravanappa et al., 2012a) in different parts of the world, till now, no cultivated okra variety or hybrid had shown absolute resistance. Alternative sources of resistance to these viral diseases may be available in wild species like *A. manihot, A. crinitus, A. angulosus*, including certain landraces of *A. tetraphyllus* (Singh et al., 2007). But, for the successful transfer of desirable resistant genes from the wild-species to the *A*. *esculentus*, the botanical distance act as a barrier. Moreover, intermediate forms resulting from natural hybridization do occur which are being used by the breeders for viral disease resistance program with very little success (Singh et al., 2007). Alternatively, all available germplasm should be evaluated under natural and artificial epiphytotic conditions to address the immediate problem of viral diseases.

Some success has been attained in transferring YVMV resistance genes from the wild-species into cultivated okra species resistant cultivars such as P7 (*Abelmoschus esculentus* × *A. manihot* ssp. manihot), Parbhani Kranti (*A. esculentus* × *A. manihot*), selections 4 and 10 (*A. esculentus* × *A. manihot* ssp. *tetraphyllus*) (Jambhale and Nerkar, 1986) have been released (Table S4). Although, more than 50 improved varieties and hybrids have been released in India, but resistance to begomoviruses is found unstable with frequent breakdowns. This may be due to the pathogenic variability, selection of okra lines which are symptomless carriers or due to the emergence of polyphagous “B” biotype of *B. tabaci* with its increased host-range. An alternative approach to controlling YVMV and OELCV incidence in okra are through the reduction of whitefly vector populations using insecticides, physical barriers, or by removal of symptomatic virus-infected plants (Table S5). However, whiteflies are showing moderate to strong resistance to various insecticides. Therefore, it is required to adopt non-conventional methods of breeding, combining biotechnological tools for the development of pre-breeding lines resistant to viral stresses (Sanwal et al., 2014).

Durable resistance to begomoviruses poses a serious challenge to both breeders and pathologists as these viruses are highly diverse, and constantly generate new forms via recombination. Consequently, use of novel biotechnological tools will help in the achieving resistance against begomovirus in okra. Identification of markers linked to the resistance genes and its pyramiding for combining multiple disease resistance genes in various backgrounds should be done. In tomato, significant progress has been made for imparting resistance against TYLCV and various resistance genes are mapped on a different chromosome. Unfortunately, same has not been yet achieved for okra, due to the paucity of genomic resources including markers linked to the begomovirus resistance genes. Additionally, identifying the linked markers to various resistance sources, will not only explain the role of these components but also enable the mapping which will lead to more durable levels of resistance (Lapidot and Friedmann, 2002). Attempts are on to incorporate broad spectrum resistance through identification of major QTLs and development of okra varieties with durable resistance to YVMV/OELCV (Sanwal et al., 2014).

The advancements in recombinant DNA technology and the optimization of transformation and regeneration protocols in okra has enabled the use of a genetic engineering approach for breeding. The pathogen-derived resistance (PDR) strategy seems very promising for the management of begomoviruses since it involves engineering resistance by transforming a susceptible plant using pathogen-derived gene sequences. The PDR can be achieved through various ways such as capsid protein-mediated resistance, movement protein-mediated resistance, defective interfering (DI) viral DNA, incorporation of genes in antisense orientation, and truncated or mutated replicase (*Rep*) (*Cl* or *ACl*) gene (Lapidot and Friedmann, 2002).

A transgenic is already developed for the okra shoot, and fruit borer (Narendran et al., 2013); thus, it is required to develop more transgenics especially using CP genes for viral diseases like YVMV and ELCV. However, okra still lacks very efficient and robust transformation protocol. In addition, viral sequences shared by several begomoviruses can be exploited for the development of pathogen-derived resistance in okra (Lapidot and Friedmann, 2002). Also, introgressed resistance genes from wild okra species may complement the transgenic plants showing only partial resistance.

The international initiative “OneKP project” is currently assembling the transcriptomes of 1,000 plant species (http://www.onekp.com/), but unfortunately, *Abelmoschus* is not a target plant of this project. While transferring the begomovirus resistance via wide-hybridization, the problem of very poor seed germination can be overcome by the optimization of the embryo-rescue protocol. In okra, whole genome sequencing appears quite complicated due to its very huge genome size (16,000 mb) spanning over 65 linkage groups in association with complex polyploidy (Sanwal et al., 2014). *A. esculentus* (2*n* = 130) is considered as the most likely amphidiploid of *A. tuberculatus* (2*n* = 58) and *A. ficulneus* (2*n* = 72) (Datta and Naug, 1968). Thus, genome sequencing of these two probable diploid ancestor species is expected to provide the desired genomic information about the *A. esculentus*. Therefore, the institutions working on okra should work together for the identification and validation of robust markers linked with resistance to begomoviruses. Further, a large number of polymorphic markers should be identified and used to develop a dense okra linkage-map, so that the linked markers can be used to screen the resistance sources and can be utilized for the development of desired breeding populations (Sanwal et al., 2014).

The tools should be developed which can aid in quick identification of the begomovirus strains associated with YVMD/OELCD. Although, Venkataravanappa and Reddy (2013) have shown the use of non-radioactive DNA probe for the routine large-scale diagnosis of geminiviruses affecting okra. But, cost-effective and user-friendly DNA-based diagnostics should be developed for the large-scale screening of the germplasms for viral disease resistance. Moreover, effective identification and validation of SNPs are relatively difficult due to the occurrence of paralogous and or homoeologous loci in okra. Therefore, various complexity reduction approaches like genotyping-by-sequencing (GBS), IIB digest restriction-site associated DNA (2b-RAD) and reduced representation libraries (RRLs) based on NGS platforms could be successfully applied (Wang et al., 2015). The okra leaf- and pod- transcriptome of resistant and susceptible genotypes is expected to provide a unique collection of a significant number of okra gene sequences and annotation of these unigene sequences is expected to serve functional genetic approaches in this amphipolyploid crop.

For begomoviruses, numerous pathogen-derived and non-pathogen-derived approaches to achieving resistance have been investigated including RNA interference (RNAi) mediated resistance for CLCuD (Sattar et al., 2013); expression of a truncated Rep gene of CLCuKoV in antisense orientation, which in cotton showed protection (Hashmi et al., 2011). RNAi was also used for imparting protection against multiple begomovirus strains in tomato (Chen et al., 2016). Recently, vATPaseA gene transgenic tobacco, delivered sufficient short interfering RNA (siRNA) to whiteflies feeding on them, leading to their mortality via significant silencing response (Thakur et al., 2014). In addition, *B. tabaci* being a phloem feeder and most of the begomoviruses are also phloem limited; thus, expressing *Tma12*, a known toxin against *B. tabaci* under the control of a phloem-specific promoter may impart resistance against both begomovirus and *B. tabaci* (Zaidi et al., 2017). Moreover, these approaches should be optimized for simultaneous incorporation of resistance against both begomoviruses and whiteflies in okra.

Another novel route to impart resistance to different begomoviruses in tomato has been reported by using an m-RNA surveillance factor Pelota, which is implicated during ribosome recycling phase of protein synthesis (Lapidot et al., 2015). So far, biotechnological approaches are targeted to control only helper begomoviruses and not for the associated satellites, which adds several functions to the helper begomoviruses. Recently, a multiplexed clustered regulatory interspaced short palindromic repeats (CRISPR)/CRISPR associated nuclease 9(Cas9) system was developed, where a cassette of sgRNA is designed to target not only the whole CLCuD-associated begomovirus complex but also the associated satellite molecules for incorporating more durable resistance in cotton (Iqbal et al., 2016). However, similar resistance approach against any okra-infecting begomoviruses has not been explored by any researcher till date. Thus, the amalgamation of “conventional/classical” breeding approaches with “non-conventional/biotechnological” approaches can identify a range of resistance genes, that can be deployed in different combinations for imparting resistance against various strains of begomovirus. An outline of begomoviruses infection and its management strategies in okra is presented in Figure 2.

## Conclusions

Historically, more than 99% of the worldwide okra cultivation has been localized in the developing countries of Asia and Africa. Until recently, very little attention has been paid to its genetic improvement, and genomic information on *Abelmoschus* is practically absent. Also, there is paucity in the development and implementation of molecular techniques in okra breeding in comparison to that available for other major crop species (Schafleitner et al., 2013). Even though the benefits of genetic engineering to small and resource-poor farmers have been demonstrated especially for the Bt cotton, but the political will to facilitate this process for other crops including okra is still very weak. Any breakthrough in the transgenic or highly linked marker(s) with viral disease resistance QTLs will pave a new way for the improvement of viral disease resistance in okra. Attempts should also be made for the integration of both coat protein genes and antisense RNA technology for the effective viral resistance in okra (Sanwal et al., 2016). Reports on the development of transgenic in okra have started coming up, and like other crops, need is to simplify the process of transgenic biosafety regulations for its immediate realization under field conditions.

Lastly, the outcomes of plant-breeding should reach the farmers as seeds of improved varieties. Presently, many okra farmers across the world do not get the seeds of improved cultivars having a certain degree of viral resistance (although no absolute viral resistance is recorded for any cultivated genotype). With current and fast emerging technologies like chromosome engineering, RNAi, marker-assisted recurrent selection (MARS) and genome-wide selection (GWS), targeted gene replacement using zinc-finger nucleases, next generation sequencing (NGS), nanobiotechnology and genome editing (CRISPR/Cas9); the future seems promising for the development of designer okra having improved features for viral disease resistance (Varshney et al., 2011). Hence, it is required to use interdisciplinary approaches to tackle the serious challenges of viral disease management in okra.

**Figure 3:**
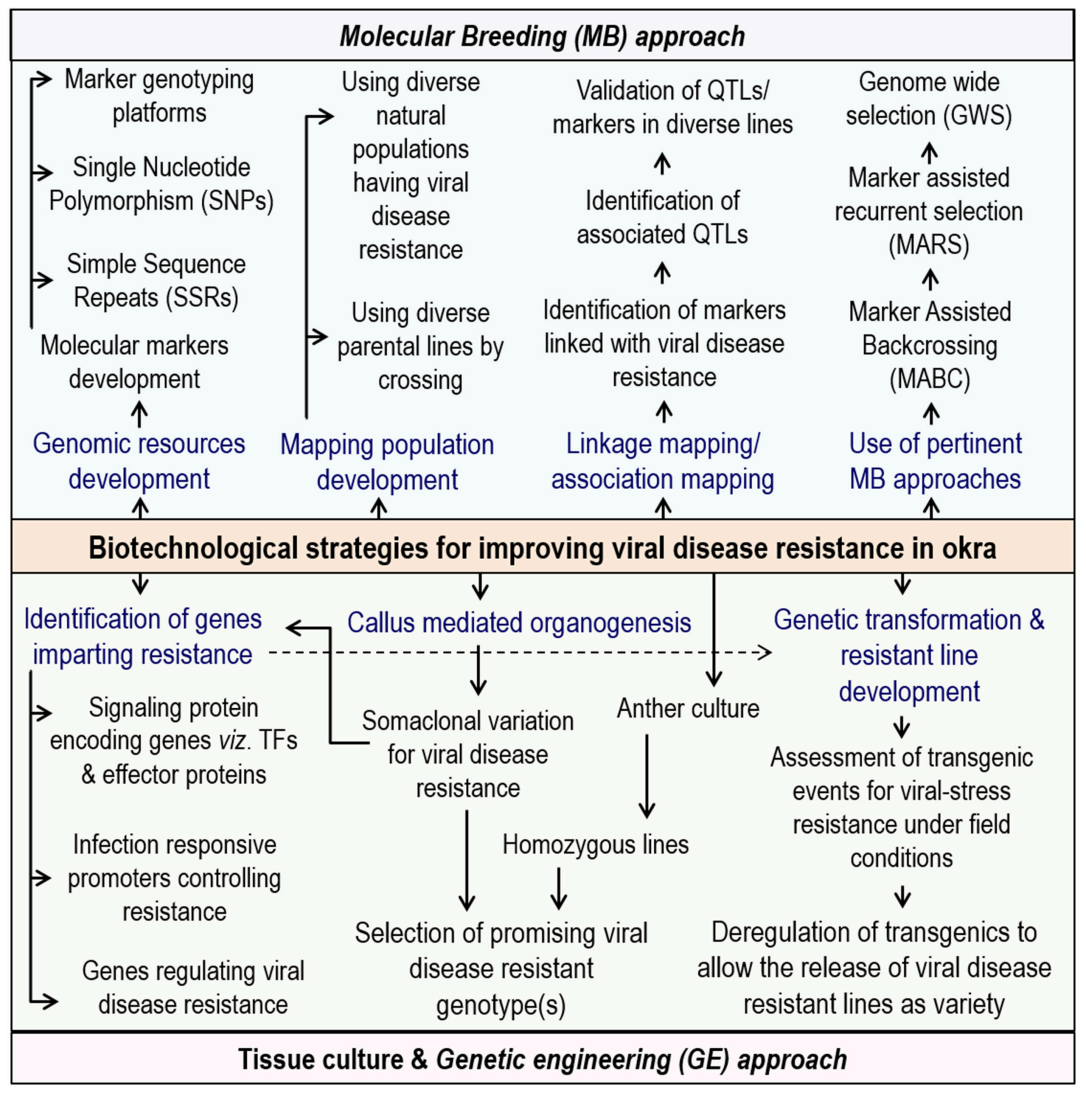
**Biotechnological strategies for improving viral disease resistance in okra**.

In addition, more funding for the integrated biotechnological approaches like marker assisted breeding, genetic engineering methodologies along with novel genomics in tandem with conventional breeding in okra will surely lead to the development of more lines having elevated resistance to the various begomoviruses (Figure 3). Also, a well-planned and long-term public sector investment for genomic research activities, jointly by India and other major okra producing African countries are needed. Of late, a few reports from different parts of the word has started coming up on the biotechnological interventions in okra and it is expected that, in years to come, significant biotechnological developments for okra improvement is going to happen.

## Author contributions

GM, BS, and TS outlined and conceptualized the review theme. GM, BS, AS, JH, and NK reviewed the begomovirus section. GM, TS, AS, ST, and PS reviewed the biotechnological intervention section. GM, BS, TS, AS, JH, NK, ST, and PS wrote the paper.

## Funding

The funds received from Indian Council of Agricultural Research, New Delhi for the Institute project and for “Consortium Research Project on Agrobiodiversity-Okra” are duly acknowledged.

### Conflict of interest statement

The authors declare that the research was conducted in the absence of any commercial or financial relationships that could be construed as a potential conflict of interest.
