# Supplementary material for: Biotechnological Advancements and Begomovirus Management in Okra (Abelmoschus esculentus L.): Status and Perspectives
Source: Front Plant Sci. 2017 Mar 17;8:360. doi: 10.3389/fpls.2017.00360 (PMC5355441; doi:10.3389/fpls.2017.00360)
Supplement: Supplementary file 1 [file Table1.DOCX]

**TABLE S1 | Comprehensive list of SSR primers reported in okra by various workers.**

| **S. No.** | **Primer name** | **Forward** | **Reverse** | **Repetitive**  **Motif** |
| --- | --- | --- | --- | --- |
| ***Medicago truncatula* EST-SSR amplified in Okra (Julier *et al.*, 2003)** | | | |  |
| 1. | OMT-1 | TGGTGACGACATACAAGAAAAGA | CCCGGTGGTTTAGGAAGTTT | [AAC]5 |
| 2. | OMT-2 | ACCACTTCTCCATCCATCCA | AGCTTGCTGCATGAGTGCT | [AAC]6 |
| 3. | OMT-3 | CAAAGGCACTTCATCAGCAA | GTGAGCGTCAATGTTGGATG | [AAC]5+6 |
| 4. | OMT-4 | TGAAGGTCAAATTGCCAAGA | TCCTTGTTTTTGAAGGTCACG | [AAG]5 |
| 5. | OMT-5 | CGATCGGAACGAGGACTTTA | CCCCGTTTTTCTTCTCTCCT | [AAG]6 |
| 6. | OMT-6 | GAAGAAGAAAAAGAGATAGATCTGTGG | GGCAGGAACAGATCCTTGAA | [AAG]8 |
| 7. | OMT-7 | CAGTTCGGGAAGAGGACAAA | ATCCCAAACCAGGTTCTTCA | [AAG]6 |
| 8. | OMT-8 | TTCCGCCCATAGTCTTTGAC | TGAAAGGGCTTAGAGGGTTTT | [AT]10 |
| 9. | OMT-9 | GGTGGAAGGAACAACTCTGG | CCGGCATGATTAAGACACAC | [AT]16 |
| 10. | OMT-10 | CACTTTCCACACTCAAACCA | GAGAGGATTTCGGTGATGT | [TC]11 |
| 11. | OMT-11 | AAAGGTGTTGGGTTTTGTGG | AGGAAGGAGAGGGACGAAAG | [TCC]6 |
| 12. | OMT-12 | CCAGTGGCAGCTACGGTACTA | GAGACGGAGGAGAAGTTGCTT | [TCC]6 |
| 13. | OMT-13 | TGGGTTGTCCTTCTTTTTGG | GGGTGCAGAAGTTTGACCA | [TG]5 |
| 14. | OMT-14 | CAAACCATTTCCTCCATTGTG | TACGTAGCCCCTTGCTCATT | [AC]5 |
| 15. | OMT-15 | GCTGACTGGACGGATCTGAG | CCAAAGCATAAGCATTCATTCA | [AG]10 |
| 16. | OMT-16 | TTTGTGTCGAGAGATGCACA | CTTGAAACTTCAACGGCATT | [AT]5 |
| **Okra SSRs (Fougat *et al.*, 2015)*** | | |  |  |
| 17. | Okra108 | AAGAAGGAGAAGAGGGAATG | TAAACCGTCTAGGAACTCCA | 230-240 |
| 18. | Okra111 | GATGGAATTGAGAAACCAGA | TGTGTTCTTCACTCTCGTCA | 230-450 |
| 19. | Okra112 | TGATCTGTCAAAGCTCCTCT | GAAATGCTGGACAAGTTAGG | 580-600 |
| 20. | Okra113 | ACGAGAGTGAAGTGGAACTG | CTCCTCTTTCCTTTTTCCAT | 200-220 |
| 21. | Okra148 | TGCTTATTCATGCTGACCTA | AGCACTTGATATCCAAGGAA | 250-300 |
| 22. | Okra151 | AAAAGACTCATCGTGGACC | GTGATTCCCTAATCCAAACA | 320-330 |
| 23. | Okra152 | GCTCTATTGATGGCGAGTAA | AAAGTCATCCAAGGTGACAA | 130-250 |
| 24. | Okra156 | CTCTCTTTGATCAGGAGCTG | TCAAGGACCGAATTATCATC | 140-230 |
| 25. | Okra157 | CAGAAACATCTCCAACATCA | ACACTTTTCAAGGGAAACC | 200-230 |
| 26. | Okra164 | CAGAAGGTCCCTTTATTCCT | TCACCCAACCATTTCTCTAC | 170-180 |
| 27. | Okra165 | GAGCTAAACCTTGCTTTTGA | CTCTTATGACTTCGGTCCAG | 150-230 |
| 28. | Okra166 | TTCCAGTTGGAGAGGTAAGA | CTTCCATTTCATCGACTTTC | 200-400 |
| 29. | Okra167 | CGGCACTCACATTTTACATA/ | GCTGTGAAGCTCTCAAAAGT | 100-200 |
| 30. | Okra169 | CTGATTCAAAGCCTCAAGAC | ATTATCTTTCCCAAGGCAAC | 200 |
| 31. | Okra170 | TGAAAGGAGAGGTACACTGG | AGGTGACTGTGATAGATCCG | 200-310 |
| 32. | Okra174 | ATGAGCTGTTCTTTGCAGAT/ | CTCCTTTAAGAACTGGGGAT | 240-260 |
| 33. | Okra175 | CCATTTACTACCCCTTCTCC | CTCAGAATGTGTGATGATGC | 110-130 |
| 34. | Okra183 | TCCCACATCAAAGGTATTTC | ATAGCAGAGGCAATCTTTCA | 190-220 |
| **Okra SSR (Yildiz *et al.*, 2015)** | | |  |  |
| 35. | SSR9 | ACCTTGAACACCAGGTACAG | TTGCTCTTATGAAGCAGTGA | - |
| 36. | SSR54 | CGAAAAGGAAACTCAACAAC | TGAACCTTATTTTCCTCGTG | - |
| 37. | SSR56 | GGCAACTTCGTAATTTCCTA | TGAGTAAAAGTGGGGTCTGT | - |
| 38. | SSR63 | GTGTTTGAAAGGGACTGTGT | CTTCATCAAAACCATGCAG | - |
| 39. | SSR78 | CTCCGACAATTCAAGAAAAG | CACCCAATCAAGCTATGTTA | - |
| 40. | SSR52 | AACACATCCTCATCCTCATC | ACCGGAAGCTATTTACATGA | - |
| 41. | SSR64 | AAGGAGGAGAAAGAGAAGGA | ATTTACTTGAGCAGCAGCAG | - |
| 42. | SSR66 | CACCAGAATTTCCCTTTTG | ACTGTTGTTTGGCTTATGCT | - |
| 43. | SSR89 | TTTGAGTTCTTTCGTCCACT | GTATTTGGACATGGCGTTAT | - |

***** For more EST-SSR primers refer Schaﬂeitner *et al.* (2013).

**References**

Fougat, R.S., Purohit, A.R., Kumar, S., Parekh, M.J. and Kumar, M. (2015). SSR based genetic diversity in *Abelmoschus* species. *Indian J. Agr. Sci.* 85(9), 1223–1228.

Julier, B., Flajoulot, S., Barre, P., Cardinet, G., Santoni S. et al. (2003). Construction of two genetic linkage maps in cultivated tetraploid alfalfa (Medicago sativa) using microsatellite and AFLP markers. *BMC Plant Biol.* 3, 9.

Schaﬂeitner, R., Kumar, S., Lin, C.Y., Hegde, S. G. and Ebert, A. (2013). The okra (*Abelmoschus esculentus*) transcriptome as a source for gene sequence information and molecular markers for diversity analysis. *Gene* 517, 27–36.

Yildiz, M., Koçak, M., Baloch, F.S. (2015). Genetic bottlenecks in Turkish okra germplasm and utility of iPBS retrotransposon markers for genetic diversity assessment. *Genet. Mol. Res.* 14(3), 10588-10602.
