# Supplementary material for: Biotechnological Advancements and Begomovirus Management in Okra (Abelmoschus esculentus L.): Status and Perspectives
Source: Front Plant Sci. 2017 Mar 17;8:360. doi: 10.3389/fpls.2017.00360 (PMC5355441; doi:10.3389/fpls.2017.00360)
Supplement: Supplementary file 2 [file Table2.DOCX]

**TABLE S2 | Various plant materials used for the isolation of DNA in okra by different workers.**

| **S. No.** | **Plant material** | **DNA Quality** | **Reference** |
| --- | --- | --- | --- |
|  | Dark-grown seedlings | +++ | Kochko and Hamon,1990 |
|  | Leaves (fresh and frozen, autumn collected) | + | Khanuja, 1999 |
|  | Fresh leaves (used polyvinylpolypyrrolidone, PVP) | ++ | Singh and Kumar, 2012 |
|  | Fresh leaves (do not require liquid nitrogen) | +++ | Ahmed et al.*,* 2013 |
|  | Leaves (DNeasy-Qiagen DNA extraction kits) | ++ | Narendran et al.*,* 2013 |
|  | Dried leaves (do not require liquid nitrogen) | ++ | Meena et al.*,* 2014 |
|  | Fresh leaves | +++ | Adiger and Sridevi, 2014 |
| Where: + : Poor quality; ++ : Good quality; +++ : Very good quality DNA | | | |

**Reference**

Adiger, S. and Sridevi, O. (2014). Isolation of DNA from mucilage-rich okra (*Abelmoschus esculentus* L.) for PCR Analysis. *Trends Biosci.* 7(16), 2306-2309.

Ahmed, N., Nawaz, S., Iqbal, A., Mubin, M., Butt, A., Lightfoot, D.A. and Maekawa, M. (2013). Extraction of high-quality intact DNA from okra leaves despite their high content of mucilaginous acidic polysaccharides. *Biosci. Methods* 4(4), 19-22.

Khanuja, S. P., Shasany, A. K. and Kumar, S. (1999). Rapid isolation of DNA from dry and fresh samples of plants producing large amounts of secondary metabolites and essential oils. *Plant Mol. Biol. Rep.* 17, 1-7.

Kochko, A. and Hamon, S. (1990). A rapid and efﬁcient method for the isolation of restrictable total DNA from plants of the genus *Abelmoschus*. *Plant Mol. Biol. Rep*. 8, 3–7.

Meena, R. K., Chhatterjee, T. and Thakur, S. (2014). An efficient method of genomic DNA isolation from mucilage-rich okra leaves for molecular biology studies. *Indian J. Appl. Res.* 4(1), 57-59.

Narendran, M., Shirale, D., Parimi, S., Deole, S. G., Nanote, A., Char, B. R., Harkude, S., Bihani, P. and Zehr, U. B. (2013). Efﬁcient genetic transformation of okra (*Abelmoschus* *esculentus* (L.) Moench) and generation of insect-resistant transgenic plants expressing the cry1Ac gene. *Plant Cell Rep.* 32, 1191–1198.

Singh, V. and Kumar, V. (2012). An optimized method of DNA isolation from highly mucilage-rich okra (*Abelmoschus esculentus* L.) for PCR analysis. *Adv. Appl. Sci. Res*. 3(3), 1809-1813.
