# Supplementary material for: Biotechnological Advancements and Begomovirus Management in Okra (Abelmoschus esculentus L.): Status and Perspectives
Source: Front Plant Sci. 2017 Mar 17;8:360. doi: 10.3389/fpls.2017.00360 (PMC5355441; doi:10.3389/fpls.2017.00360)
Supplement: Supplementary file 3 [file Table3.DOCX]

**TABLE S3 | Sequence comparison of begomoviruses from various locations**

| **S. No.** | **Begomovirus infecting okra** | **Similarity with other Begomovirus** | **Sequence similarity** | **Reference** |
| --- | --- | --- | --- | --- |
|  | Okra Enation Leaf Curl Virus (OELCuV, India) | Cotton Leaf Curl Virus Burewala (CLCuVBur3, Pakistan) | ~99% | Hameed et al., 2014 |
|  | OELCuV (Haryana, India) | Mesta Yellow Vein Mosaic Virus (MeYVMV, India) | 84 to 87% | Venkataravanappa et al., 2015 |
|  | Okra yellow vein Bhubhaneswar virus (OYBHU, Bhubaneshwar, India) | CYVMV (India) | 81 to 86% | Venkataravanappa et al., 2013b |
|  | YVMV (Karnataka, India) | BYVMaV (Bhendi Yellow Vein Maharastra Virus) (Maharastra, India) | ~90% | Venkataravanappa et al., 2016 |
|  | BYVMaV (Maharastra, India) | BYVMV (India) | ~89% | Venkataravanappa et al., 2016 |
|  | YVMV (India) | Cotton Leaf Curl Gemini Virus (India) | ~95% | Venkataravanappa et al., 2013a |

**References**

Hameed, U., Zia-Ur-Rehman, M., Herrmann, H.-W., Haider, M. S. and  Brown, J. K. (2014). First report of *okra enation leaf curl virus* and associated cotton leaf curl multan betasatellite and cotton leaf curl multan alphasatellite infecting cotton in Pakistan: A new member of the cotton leaf curl disease complex. *Plant Dis*. 98(10), 1447. doi:10.1094/PDIS-04-14-0345-PDN

Venkataravanappa, V., Reddy, C. N. L., Chauhan, N. S., Singh, B., Sanwal, S. K., Reddy, M. K. (2016). Nucleotide sequencing and an improved diagnostic for screening okra (*Abelmoschus esculentus* L.) genotypes for resistance to a newly described begomovirus in India. *J. Hort. Sci. Biotechnol*. doi:10.1080/14620316.2015.1123407

Venkataravanappa, V., Reddy, C. N. L., Devaraju, A., Jalali, S., Reddy, M. K. (2013a). Association of a recombinant Cotton leaf curl Bangalore virus with yellow vein and leaf curl disease of okra in India. *Indian J. Virol.* doi:10.1007/s13337-013-0141-4.

Venkataravanappa, V., Reddy, C. N. L., Jalali, S., Reddy, M. K. (2013b). Molecular characterization of a new species of begomovirus associated with yellow vein mosaic of bhendi (okra) in Bhubhaneswar, India. *Eur. J. Plant Pathol*. 136, 811–822. doi:10.1007/s10658-013-0209-4

Venkataravanappa, V., Reddy, C.N. L., Jalali, S., Briddon, R. W. and Reddy, M. K. (2015). Molecular identification and biological characterization of a begomovirus associated with okra enation leaf curl disease in India. *Eur. J. Plant Pathol*. 141, 217–235. doi:10.1007/s10658-014-0463-0
