# Supplementary material for: Biotechnological Advancements and Begomovirus Management in Okra (Abelmoschus esculentus L.): Status and Perspectives
Source: Front Plant Sci. 2017 Mar 17;8:360. doi: 10.3389/fpls.2017.00360 (PMC5355441; doi:10.3389/fpls.2017.00360)
Supplement: Supplementary file 4 [file Table4.DOCX]

**TABLE S4 | Sources of resistance to begomoviruses in okra.**

| **S.**  **No.** | **Wild / Cultivated genotypes** | **Country** | **Disease resistance** | **Reference** |
| --- | --- | --- | --- | --- |
|  | *A. tuberculatus*, *A. angulosus*, *A. manihot*, IC-1542 | India | Symptomless carrier | Nariani and Seth, 1958 |
|  | EC-31830, Asuntem Koko from Ghana [*Abelmoschus manihot* (L) Medicus ssp. *Manihot*] | India | Immune to YVMV | Sandhu *et al.*, 1974 |
|  | *A. manihot* (L.) Medik. ssp. manihot | India | Symptomless carrier | Thakur, 1976 |
|  | *A. manihot* group | India | Resistant to YVMD | Arumugam and Muthukrishnan, 1978 |
|  | *A. manihot* | India | Resistant to YVMD | Singh and Thakur, 1979 |
|  | 'Ghana' | India | Resistant with higher phenols, orthodihydroxy phenols and total chlorophyll content | Sharma *et al.*, 1981 |
|  | *A. manihot* ssp. manihot | India | Resistant to YVMD | Sharma and Sharma, 1984 |
|  | Germplasm lines | Nigeria | Resistant to OELCV | Atiri and Fayoyin, 1989 |
|  | Punjab Padmini and EMS-8 | India | Free from YVMD | Arora *et al.*, 1992 |
|  | Pb7, GOH6, GOH4, AROH1, Pb57 and 74 | India | Highly resistant to YVMD | Borah *et al.*, 1992 |
|  | Punjab Padmini and Punjab-7 | India | Resistant to YVMD | Sharma *et al.*, 1993 |
|  | Prabhani Kranti | India | Highly Resistant to YVMD | Dhankhar *et al.*, 1996 |
|  | P7 | India | Moderately resistant to YVMD | Dhankhar *et al.*, 1996 |
|  | Arka Anamika and Arka Abhay | India | Resistant to YVMD | Borah *et al.*, 1992; Sannigrahi and Choudhury, 1998 |
|  | Ok No. 6, LORM 1, VRO 3 and Punjab 7 | India | Free from YVMD | Batra and Singh, 2000 |
|  | VRO 4 | India | Mild reaction of YVMD | Batra and Singh, 2000 |
|  | OK-292 and OK-285 | Bangladesh | Resistant to YVMD | Rashid *et al.*, 2002 |
|  | OK-315, OK-316 and OK-317 | Bangladesh | Tolerant to YVMD | Rashid *et al.*, 2002 |
|  | Twelve gamma-ray irradiated M4 mutant lines | Thailand | Resistant to YVMD | Phadvibulya *et al.*, 2004 |
|  | Twenty-four West African accessions of cultivated okra | Bangladesh | Immune to YVMD | Abdul *et al.*, 2004 |
|  | *A. manihot* ssp. *manihot, A. manihot* ssp. *manihot* var. Ghana and West African okra | India | Symptomless carriers of YVMD | Dhankhar and Mishra, 2004 |
|  | IC218887, IC69286 and EC305619 | India | Resistant to YVMD | Abdul *et al.*, 2004 |
|  | Surkh Bhindi | Pakistan | Resistant to YVMD | Ali *et al.*, 2005 |
|  | Subz Pari and Safal | Pakistan | Moderately resistant to YVMD | Ali *et al.*, 2005 |
|  | *A. manihot, A. angulosus, A. crinitus, A. vitifolius, A. tuberculatus, A. panduraeformis, A. pungens* and *A. tetraphyllus* | India | Resistant to YVMD | Dhankhar and Mishra, 2004; Singh *et al.*, 2007 |
|  | *A. angulosus* | India | Completely free from YVMV | Prabu *et al.*, 2007 |
|  | *A. tetraphyllus, A. moschatus*, *A. caillei, A. manihot* spp. *tetraphyllus* | India | Highly resistant to YVMD | Prabu *et al.*, 2007 |
|  | *A. manihot* (L.) Medikus and *A. manihot* spp. *manihot* | India | Resistant to YVMD | Prabu *et al.*, 2007 |
|  | *A. manihot, A. crinitus*, *A. angulosus,* including certain landraces of *A. tetraphyllus* | India | Resistant to YVMD | Singh *et al*., 2007 |
|  | Arka Abhey, Arka Anamika, 50M-150, TC-17, P-7, NOH-147, NOL-101, NOH-15, ZCH-3002 and US-7109 | India | Resistant to OYVMV | Mehra et al., 2008 |
|  | KN–OYV–03 | Thailand | Moderately resistant to YVMD | Benchasri, 2011 |
|  | NOL-285 | India | Highly resistant to YVMD | Deshmukh *et al.*, 2011 |
|  | Nun-1145 and Nun-1144 | India | Moderate resistance to YVMD | Venkataravanappa *et al.*, 2012 |
|  | Saloni F_1_ | Pakistan | Highly resistant to YVMV | Ali et al., 2012 |
|  | Subz Pari | Pakistan | Moderately resistant to YVMV | Ali et al., 2012 |
|  | VRO-6 | India | Resistant to YVMV | Tiwari et al., 2012 |
|  | VRO-3 and HRB 9-2 | India | Moderately resistant to YVMV | Tiwari et al., 2012 |
|  | Deb-401’ and ‘Barsha Laxmi’ | India | Resistant to YVMV | Das et al., 2013 |
|  | VRO-109, VRO-104, VROB-178, 307 10-1 and No. 315 | India | Completely free from YVMD | Sanwal *et al.*, 2014 |
|  | Ebi Ogwu, Ojo ogwu, Tongolo, VLO, Oru ufie and Ogolo cultivars | Nigeria | Resistant to OMVD and OLCD | Sergius and Esther, 2014 |
|  | IC582757 (*A. enbeepeegeerense*) NIC5952 (*A. moschatus*), Jpn/N-2176 (*A. manihot*) IC-90340 (*A. tuberculatus*) and IIVR-Tube-1 (*A. tuberculatus*) | India | Resistant to YVMD | Sanwal *et al.*, 2014 |
|  | BCO-1, *A. caillei*, *A. manihot*, 11/RES-6, VNR Green and 12/RES-2 | India | Resistant to YVMD | Seth et al., 2016; |
|  | Arka Anamika, H-8 and H-10 | Pakistan | Resistant to YVMD | Chaudhary et al., 2016 |
|  | Soumya F_1_ and Reshma | Pakistan | Moderately resistant to YVMD | Chaudhary et al., 2016 |
|  | ‘Tulasi’ and ‘Trisha’ | India | Moderately resistant to YVMD | Venkataravanappa *et al*., 2016 |
|  | BCO-1 and VNR Green | India | Resistant to YVMD | Seth et al., 2017 |

**Reference**

Abdul, N. M., Joseph, J. K. and Karuppaiyan, R. (2004). Evaluation of okra germplasm for fruit yield, quality and field resistance to yellow vein mosaic virus. *Indian J. Plant Genet. Res.* 17, 241–4.

Ali, M. I., Khan, M. A., Rashid, A., Ehetisham-ul-haq, M., Javed, M. T. and Sajid, M. (2012). Epidemiology of Okra Yellow Vein Mosaic Virus (OYVMV) and its management through tracer, mycotal and imidacloprid. *Am. J. Plant Sci.* 3, 1741-1745. doi:10.4236/ajps.2012.312212

Ali, S., Khan, M. A., Habib, A., Rasheed, S. and Iftikhar, Y. (2005). Correlation of environmental conditions with okra yellow vein mosaic virus and *Bemisia* *tabaci* population density. *Int. J. Agric. Biol.* 7, 142–144.

Arora, S. K., Dhanju, K. C. and Sharma, B. R. (1992). Resistance in okra [*Abelmoschus esculentus* (L.) Moench] genotypes to yellow vein mosaic virus. *Plant Dis. Res.* 7, 221–225.

Arumugam, R. and Muthukrishnan, C. R. (1978). Nitrogenous compounds in relation to resistance to yellow vein mosaic disease of okra. *Progressive Hort.* 10, 17–21.

Atiri, G. I. and Fayoyin, G. A. (1989). Horizontal resistance to okra leaf curl virus in okra germplasm. *An. App. Biol.* 11(4), 152-153.

Batra, V.K. and Singh, J. (2000). Screening of okra varieties to yellow vein mosaic virus under field conditions. *Veg. Sci.* 27, 192–193.

Benchasri, S. (2011). Screening for yellow vein mosaic virus resistance and yield loss of okra under field conditions in Southern Thailand. *J. Animal Plant Sci.* 12(3), 1676-1686.

Borah, G.C., Saikia, A.K. and Shadeque, A. (1992). Screening of okra genotypes for resistance to yellow vein mosaic virus disease. *Indian J. Virol*. 8:55–57

Chaudhary, A., Khan, M.A. and Riaz, K. (2016). Spatio-temporal pattern of okra yellow vein mosaic virus and its vector in relation to epidemiological factors. *J. Plant Pathol. Microbiol.* 7, 360. doi:10.4172/2157-7471.1000360

Das, S., Chattopadhyay, A., Dutta, S., Chattopadhyay, S.B. and Hazra, P. (2013). Breeding okra for higher productivity and yellow vein mosaic tolerance. *Int. J. Veg. Sci.* 19(1), 58-77. <http://dx.doi.org/10.1080/19315260.2012.675024>

Deshmukh, N. D., Jadhav, B. P., Halakude, I. S. and Rajput, J. C. (2011)*.* Identification of new resistant sources for yellow vein mosaic virus disease of okra (*Abelmoschus esculentus* L.). *Veg. Sci.* 38(1), 79–81.

Dhankhar, B.S. and Mishra, J. P. (2004). Objectives of okra breeding. (*In*) Hybrid Vegetable Development, pp 195–209. Singh, P. K., Dasgupta, S. K. and Tripathi. S. K. (Eds). Haworth Press, Binghamton, NY.

Dhankhar, S.K., Dhankhar, B.S. and Saharan, B.S. (1996). Screening of okra genotype for resistance to yellow vein mosaic disease. *Ann. Biol.* 12, 90–92.

Mehra, R., Dhawan, P. and Batra, V. (2008). Screening of okra germplasm against okra yellow vein mosaic virus and okra leaf curl virus diseases for sustainable cultivation. *Haryana J. Hortic. Sci.* 37(l&2), 12l-122.

Nariani, T.K. and Seth, M.L. (1958). Reaction of *Abelmoschus* and *Hibiscus* species to yellow vein mosaic virus. *Indian Phytopathol.* 11, 137–140.

Phadvibulya, V., Puripanyavanich, V., Adthalungrong, A., Kittipakorn, K. and Lavapaurya, T. (2004). Induced mutation breeding for resistance to yellow vein mosaic virus in okra., In: Proceedings of a Final Research Coordination Meeting organized by the Joint FAO/IAEA Division of Nuclear Techniques in Food and Agriculture; 2003 May 19–23; Pretoria (South Africa) p. 155–175.

Prabu, T., Warade, S. D. and Ghante, P. H. (2007). Resistance to okra yellow mosaic virus in Maharashtra. *Veg. Sci.* 34(2), 119–122.

Rashid, M. H., Yasmin, L., Kibria, M. G., Mollik, A. K. M. S. R. and Hossain, S. M. (2002). Screening of okra germplasm for resistance to yellow vein mosaic virus under field conditions. *Plant Pathol. J.* 1, 61–62.

Sandhu, G. S., Sharma, B. R., Singh, B. and Bhalla, J. S. (1974). Sources of resistance to jassids and white fly in okra germplasm. *Crop Improvement* 1, 77–81.

Sannigrahi, A.K. and Choudhury, K. (1998). Evaluation of okra cultivars for yield and resistance to yellow vein mosaic virus in Assam. *Environ. Ecol.* 16, 238–239.

Sanwal, S. K., Singh, M., Singh, B. and Naik, P. S. (2014). Resistance to yellow vein mosaic virus and okra enation leaf curl virus: challenges and future strategies. *Curr. Sci*. 106, 470-1471.

Sergius, U. O. and Esther, D. U. (2014). Screening of *Abelmoschus esculentus* and *Abelmoschus callei* cultivars for resistance against okra leaf curl and okra mosaic viral diseases, under field conditions in South Eastern Nigeria. *Afr. J. Biotechnol*. 13(48), 4419-4429. doi:10.5897/AJB2014.13686.

Seth, T., Chattopadhyay, A., Chatterjee, S., Dutta, S., Singh, B. (2016). Selecting parental lines among cultivated and wild species of okra for hybridization aiming at YVMV disease resistance. *J. Agr. Sci. Tech.* 18, 751-762.

Seth, T., Chattopadhyay, A., Dutta, S., Hazra, P. and Singh, B. (2017). Genetic control of yellow vein mosaic virus disease in okra and its relationship with biochemical parameters. *Euphytica* 213, 30. doi:10.1007/s10681-016-1789-9

Sharma, B.R., Arora, S.K., Dhanju, K.C. and Ghai, T.R. (1993). Performance of okra cultivars in relation to yellow vein mosaic virus and yield. *Indian J. Virol.* 9, 139–142.

Sharma, B.R., Kumar, V. and Bayay, K.L. (1981). Biochemical basis of resistance to yellow vein mosaic virus in okra. *Genet. Agraria* 35, 121–130.

Sharma, B.R. and Sharma, O.P. (1984). Field evaluation of okra germplasm against yellow vein mosaicvirus. *Punjab Hort. J.* 24, 131–133.

Singh, M. and Thakur, M.R. (1979). Nature of resistance to yellow vein mosaic in *Abelmoschus manihot* spp. *manihot*. *Curr. Sci.* 48, 164–165.

Singh, B., Rai, M., Kalloo, G., Satpathy, S. and Pandey, K. K. (2007). Wild taxa of okra (Abelmoschus species): Reservoir of genes for resistance to biotic stresses. *Acta Horticulturae* 752, 323–328.

Thakur, M.R. (1976). Inheritance of resistance to Yellow Vein Mosaic (YVM) in a cross of okra species, *Abelmoschus esculentus* *A. manihot* ssp. manihot. *SABRAO J.* 8, 69–73.

Tiwari, A., Singh, B., Singh, T.B., Sanval, S.K. and Pandey S.D. (2012). Screening of okra varieties for resistance to yellow vein mosaic virus under field condition. *HortFlora Res. Spect.* 1(1), 92-93.

Venkataravanappa, V., Reddy, C. N. L. and Reddy, M. K. (2012). Begomovirus characterization, and development of phenotypic and DNA-based diagnostics for screening of okra genotype resistance against bhendi yellow vein mosaic virus. *3 Biotech*. 3(6), 461–470. doi:10.1007/s13205-012-0107-z
