# Supplementary material for: Biotechnological Advancements and Begomovirus Management in Okra (Abelmoschus esculentus L.): Status and Perspectives
Source: Front Plant Sci. 2017 Mar 17;8:360. doi: 10.3389/fpls.2017.00360 (PMC5355441; doi:10.3389/fpls.2017.00360)
Supplement: Supplementary file 5 [file Table5.DOCX]

**TABLE S5 | Various viral disease management practices reported in okra.**

| **S. No.** | **Country** | **Disease** | **Management practice** | **Reference** |
| --- | --- | --- | --- | --- |
|  | India | YVMV | Spraying with 2% emulsiﬁable mineral oil | Nene, 1973 |
|  | India | YVMV | Insecticidal spray and 1% mineral oil spray at 10-days-interval | Sastry and Singh, 1973 |
|  | Nigeria | YVMV and OELCV | Dieldrin (0.12%), DDT (0.18%) + Lindane (Didigam) and monocrotophos (0.12%) applied at weekly intervals as foliar sprays. | Ewete, 1974 |
|  | India | YVMV | Resistance genes introgression from the wild species | Arumugam et al., 1975 |
|  | Nigeria | OELCV | Insecticidal spray | Lana, 1976 |
|  | India | YVMV | Border cropping with maize | Singh et al., 1979 |
|  | India | YVMV | Early sowing of okra during *kharif* season especially in Northern India | Gill et al., 1982 |
|  | India | YVMV | Soil application of methyl phosphoro-dithioate | Khan and Mukhopadhyay, 1985 |
|  | India | YVMV | Introgression of resistance genes from the wild-species | Thakur, 1986 |
|  | India | YVMV | Introgression of resistance genes from the wild-species in to cultivated okra | Jambhale and Nerkar, 1986 |
|  | Nepal | YVMV | Insecticidal spray | Dahal et al., 1992 |
|  | India | YVMV | leaf extracts of *Prospos chilensis* and *Bougainvillea spectabilis* | Pun et al., 1999 |
|  | Pakistan | OYVMV | Imidacloprid spray | Ali et al., 2000 |
|  | India | YVMV | Spraying the pearl millet barrier crop with Achook (a neem product) | Kalita, 2003 |
|  | India | YVMV | Maize as border crop | Pun et al., 2005 |
|  | India | YVMV | Spraying with neem oil and neem kernel extracts | Pun et al., 2005 |
|  | Pakistan | OYVMV | Neem extract and Effective microbes | Ali et al., 2005 |
|  | India | YVMV | Extracts of neem (*Azadiracta indica*) fruits, garlic (*Allium sativum*) bulbs, karamja (*Pongamia pinnata*) leaves and mehogoni (*Swietenia macrophylla*) seeds | Bhyan et al., 2007 |
|  | India | OYVMV | Aqueous root extract of *Boerhaavia diffusa* as foliar sprays at concentration of 4% at 7 days interval | Singh et al., 2009 |
|  | Burkina Faso | OLCD | improved commercial okra cultivars | Tiendrébéogo et al., 2010 |
|  | India | YVMV | Neem products and insecticides spray | Venkataravanappa et al., 2011 |
|  | Nigeria | OLCV | Carbaryl + ash | Askira, 2011 |
|  | India | YVMV | Diafenthiuron 50 WP @ 600 g/ha, imidacloprid 70 WG @ 75 g/ha and thiamethoxam 25 WG @ 100 g/ha treatments | Ghosal and Chatterjee, 2013 |
|  | Ghana | OLCD | 10% (w/v) crude extract each of neem leaf, garlic, mahogany bark, chili pepper fruit, pawpaw dried leaf, bougainvillea leaf | Asare-Bediako et al., 2014 |
|  | India | YVMV | Foliar spray of partially clarified aqueous root extract of *Boerhaavia diffusa* (4%) at 7 days interval | Dubey and Singh, 2015 |

**References**

Ali, S., Khan M.A., Habib A., Rasheed S. and Iftikhar Y. (2005). Management of yellow vein mosaic disease of okra through pesticide/bio-pesticide and suitable cultivars. *Int. J. Agril. Biol.* 7(1), 145–147.

Ali, M., Hossain, M. Z. and Sarker, N. C. (2000). Inheritance of yellow vein mosaic virus (YVMV) tolerance in a cultivar of okra (*Abelmoschus esculentus* (L.) Moench). *Euphytica* 111, 205–209.

Arumugam, R., Chelliah, S. and Muthukrishnan, C. R. (1975). Abelmoschus manihot: a source of resistance to bhindi yellow vein mosaic. *Madras Agril. J.* 62, 310–312.

Asare-Bediako, E., Addo-Quaye, A. and Bi-Kusi, A. (2014). Comparative efficacy of plant extracts in managing whitefly (*Bemisia tabaci* Gen) and leaf curl disease in okra (*Abelmoschus esculentus* L). *Am. J. Agril. Sci. Technol.* 2(1), 31-41. doi:10.7726/ajast.2014.1004

Askira, A.B. (2011). A survey on the incidence of okra leaf curl virus on okra in Lake Alau area of Borno State, Nigeria. *Pan-African J.*  4(1), 122-127.

Bhyan, S. B., Alam, M.M. and Ali, M.S. (2007). Effect of plant extracts on okra mosaic virus incidence and yield related parameters of okra. *Asian J. Agril. Res.* 1(3), 112.

Dahal, G., Neupane, F.P. and Baral, D.R. (1992). Effect of planting and insecticides on the incidence and spread of yellow vein mosaic of okra in Nepal. *Int. J. Plant Dis.* 10, 109–124.

Dubey, R.K. and Singh, A.K. (2015). Plant protection & biological control of viral infection on okra (*Abelmoschus esculentus*) by root extract of *Boerhaavia diffusa*. *Int. J. Innovative Res. Sci. Eng. Technol.* 4(5), 3777-3781. doi:10.15680/IJIRSET.2015.0405137

Ewete, M. F. (1974). The effect of insecticidal pest control management on seed yield and viability in okra (*Abelmoschus esculentus*) (L.1 (Moench). Diploma in Plant Protection Thesis, University of Ibadan. 105 pp.

Gill, S.S., Singh, H. and Sandhu, K. S. (1982). Effect of sowing dates and plant density on the incidence of yellow vein mosaic virus on seed crop of okra (*Abelmoschus esculentus* L. Moench). *Indian J. Ecol.* 9, 160–162

Ghosal A. and Chatterjee M. L. (2013). Management of okra yellow vein mosaic virus by chemical control of *Bemisia tabaci* Gennadius *Indian J. Entomol.* 75(3), 236-238.

Jambhale, N. D. and Nerkar, Y. S. (1981). Inheritance of resistance to okra yellow vein mosaic disease in interspeciﬁc crosses of *Abelmoschus*. *Theor. Appl. Genet.* 60, 313–316.

Kalita, M. K. (2003). Epidemiology and management of yellow vein mosaic and leaf curl begomovirus diseases of okra. Ph.D. thesis, CCS Haryana Agricultural University, Hisar, 129 pp

Khan, M.A. and Mukhopadhyay, S. (1985). Effect of different pesticide combinations on the incidence of yellow vein mosaic virus disease of okra (Abelmoschus esculentus) and its whiteﬂy vector Bemisia tabaci Genn. *Indian J. Virol.* 1, 147–151.

Lana, A. F. (1976). Mosaic virus and leaf curl disease of okra in Nigeria. *Pest Articles and News Summaries,* 22, 474-478.

Nene, Y.L. (1973). Control of *Bemisia tabaci* Genn: a vector of several plant viruses. *Indian J. Agric. Sci.* 43, 433–436.

Pun, K.B., Doraiswamy, S. and Jeyarajan, R. (2005). Management of Okra yellow vein mosaic virus disease and its whiteﬂy vector. *Indian J. Virol.* 16, 32–35.

Pun, K.B., Sabitha, D., Jeyaran, R., Doraiswammy, S. (1999). Screening of plant species for presence of antiviral principles against okra yellow vein mosaic virus. *Indian Phytopath.* 52, 221–223.

Sastry, K.S.M. and Singh, S.J. (1973). Restriction of yellow vein mosaic virus spread in okra through the control of vector, whiteﬂy *Bemisia tabaci*. *Indian J. Mycol. Plant Pathol.* 3, 76–80.

Singh, S.J., Sastry, K.S.M. and Sastry, K.S. (1979). Effect of barrier cropping combined with insecticidal sprays on the control of yellow vein mosaic virus of okra. In: Proceedings of the symposium retrospect and prospects of research in Botany in India, 24–26 Feb, University of Gorakhpur, India.

Singh, A.K., Najam, Verma, H.N. and Awasthi, L.P. (2009). Control of natural virus infection on okra (*Abelmoschus esculentus*) by root extract of *Boerhaavia diffusa*. *Int. J. Plant Prot.* 2(2), 195-198.

Thakur, M. R. (1986). Breeding for disease resistance in okra. *Veg. Sci*. 13, 310–315.

Tiendrébéogo, F., Lefeuvre, P., Hoareau, M., Villemot, J., Konate, G., Traoré, A.S., et al. (2010). Molecular diversity of *Cotton leaf curl Gezira virus* isolates and their satellite DNAs associated with okra leaf curl disease in Burkina Faso. *Virol. J.* 7, 48.

Venkataravanappa, V., Krishnareddy, M., Lakshinimarayanreddy, C. N. and Jalali, S. (2011). Management of Okra YVM disease through neem products and insecticides. *Ann. Pl. Protec. Sci.* 19(2), 487-488.
